# Limiting Factors for Mapping Corpus-Based Semantic Representations to Brain Activity

**DOI:** 10.1371/journal.pone.0057191

**Published:** 2013-03-19

**Authors:** John A. Bullinaria, Joseph P. Levy

**Affiliations:** 1 School of Computer Science, University of Birmingham, Birmingham, United Kingdom; 2 Department of Psychology, University of Roehampton, London, United Kingdom; The University of Plymouth, United Kingdom

## Abstract

To help understand how semantic information is represented in the human brain, a number of previous studies have explored how a linear mapping from corpus derived semantic representations to corresponding patterns of fMRI brain activations can be learned. They have demonstrated that such a mapping for concrete nouns is able to predict brain activations with accuracy levels significantly above chance, but the more recent elaborations have achieved relatively little performance improvement over the original study. In fact, the absolute accuracies of all these models are still currently rather limited, and it is not clear which aspects of the approach need improving in order to achieve performance levels that might lead to better accounts of human capabilities. This paper presents a systematic series of computational experiments designed to identify the limiting factors of the approach. Two distinct series of artificial brain activation vectors with varying levels of noise are introduced to characterize how the brain activation data restricts performance, and improved corpus based semantic vectors are developed to determine how the word set and model inputs affect the results. These experiments lead to the conclusion that the current state-of-the-art input semantic representations are already operating nearly perfectly (at least for non-ambiguous concrete nouns), and that it is primarily the quality of the fMRI data that is limiting what can be achieved with this approach. The results allow the study to end with empirically informed suggestions about the best directions for future research in this area.

## Introduction

Knowledge of how brains encode and process information is of practical importance for many fields, ranging from philosophy and psychology to neuroscience and artificial intelligence. There have already been many studies by neuroscientists that have sought to explore how the brain represents semantics as patterns of neural activity in different brain areas (e.g., [1]–[5]), and related work has shown how high-level knowledge of visual objects can be reflected in patterns of individual voxel activations (e.g., [6], [7]). Recently, Mitchell et al. [8] have suggested refining our understanding of how the human brain encodes semantic knowledge by mapping independent computational representations of lexical semantics for particular concrete objects to corresponding patterns of brain activation as measured by fMRI. In principle, any reliable semantic representation could be used as the inputs for those models, but computational linguists have already established that surprisingly good representations of lexical semantics can be generated from the word co-occurrence statistics of large text corpora (e.g., [9]–[13]), so those are a natural choice. This led Mitchell et al. to train linear regression models to predict brain activations from corpus derived semantic representations for 60 concrete nouns (5 from each of 12 semantic categories such as *insects*, *tools*, *vegetables, vehicles*), achieving generalization performance levels significantly above chance [8].

That study has already been the subject of much further investigation, and numerous variations of the original prediction model have been suggested (e.g., [14]–[20]). Our own study [18] used improved general purpose corpus-based semantic representations [12], [21] with the original fMRI data to achieve the best performance so far on the brain activation prediction task. However, even the best of those results have only provided limited improvement over the original study, the performance levels are still not good enough for reliable conclusions to be deduced, and it is not obvious what factors are limiting progress. Since the idea of relating patterns of brain activation to representations of semantics is becoming increasingly widespread [22], understanding what is limiting progress in this area will be of considerable general interest. This paper begins to explore whether the current poor performance is due to noise or deficiencies in the fMRI brain activation vectors, or in the semantic input vectors, or in the learned mappings, or in some combination of all three. That is done by first using an independent measure of semantic vector quality to identify where the biggest problems may be, then testing the linear mapping approach on a range of artificial brain activation vectors that includes many which are much cleaner than those feasible using existing brain imaging technology, and finally exploring the effect of using improved semantic representations for the inputs. It will test right up to the limiting cases, determining how well the current brain activation vectors might perform given perfect corpus-based semantic representations, how well the current corpus-based semantic representations might do given perfect brain activation vectors, and how much training data is required for the current linear model approach to work well given highly consistent inputs and outputs. Of course, the model and data interact, and the performance on the existing data could potentially be improved by having a better model that can capture more of the signal that might be present. The best we can do with an empirical approach is study the best model we currently have, but we do need to bear in mind that the limiting factors in the data may well change if better models can be developed.

## Methods and Results

The original Mitchell et al. study [8] involved eliciting brain activations corresponding to each of 60 concrete nouns by asking a series of healthy participants to mentally generate a set of properties six times for each object when presented with previously studied word-picture pair stimuli for those objects. Then data from each individual participant for each of the 1770 combinations of 58 out of the full set of 60 words were used to fit a linear regression model that maps the input corpus-derived semantic representations to the associated patterns of brain activation, and each model was tested on its ability to generalize to predict the activations of the two held-out words. In total, a set of 1770 prediction models was created for each of nine participants, and the average prediction performance was computed. Performance in the current study is measured using exactly the same leave-two-out brain activation prediction task. Our previous systematic study [18] has shown that, in addition to using improved input semantic representations, better results can also be obtained by including a standard (ridge regression type) regularization in the linear model, with the regularization parameter and number of output voxels optimized for each type of input and output representation. If, for each word *i*, the vector of input features is *f_i_*, the vector of brain activations is *a_i_*, and the vector of model outputs is *m_i_*, the models' computations can conveniently be expressed as the minimization of the sum-squared output error *E* of the model over the set of training items *i* with regularization parameter *λ*: 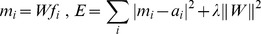



and the matrix *W* of model weights/coefficients can easily be computed using standard matrix pseudo-inversion techniques. That approach will be adopted without variation as the standard prediction task model throughout this study.

In the original Mitchell et al. study [8], a model was deemed to have made a correct prediction if the sum of the cosine distances between the predicted and measured brain activation patterns for the two withheld words was smaller than that with the two words' predictions switched. The fraction of correct predictions in that sense will here be called the pair performance *PairPerf*. As noted previously [18], computing and comparing each of the individual cosine distances, rather than the sum of the pair, gives a better cross-validated estimate of the average probability that the model's output for a given word is closer to the correct word target output than that of any other word. The fraction of correct predictions in that sense will be called the performance *Perf*. To facilitate comparisons with other studies, results for both performance measures will be presented throughout this paper. For both measures, chance performance is 0.5 and perfect performance is 1.0, but *PairPerf* is generally higher than *Perf* at intermediate levels. Empirical permutation tests show that the 0.05 statistical significance level falls at 0.58 for *Perf* and 0.62 for *PairPerf*. The relative reliability of these two measures will be considered in more detail later, once we have concrete results to analyse.

It is worth noting at this point that any set of words could be used for this kind of prediction task, and that the semantic category structure of the chosen 60 words is not crucial for it. Human cognition is highly capable of operating in a noisy world where category boundaries are much more imprecise and shifting, and we know that the representations that brains use are far more subtle than what is captured by the simple category structure used here. This is one of the reasons why we believe that lexical co-occurrence statistics provide a particularly useful basis for models of conceptual structure, because, whether or not semantic memory is learned directly from language exposure, these statistics reflect the real-world linguistic usage of concrete concepts, and may thus be able to capture some of the complexity of the semantic structure inherent in cortical semantic representations. For the purposes of the models studied in this paper, however, the chosen simplified category structure *is* useful in that it provides a straightforward indicator of the difficulty of the task for particular withheld word pairs – words from different semantic categories will naturally be easier to distinguish than words from within the same category. It also enables the definition of a simple independent measure of the reliability of the associated semantic representations.

A standard approach for measuring the quality of semantic representations involves applying a general-purpose clustering algorithm to the semantic vectors for a particular set of *n* words, and computing the purity of the resulting clusters using the known semantic categories for each word [21]. The purity *P_r_* of a given cluster *r* is the fraction of its members that belong to the most represented category within that cluster, and the overall purity *P* of clustering is the weighted average of all the individual cluster purities *P_r_*. So, 
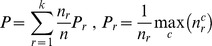



where *n_r_* and *n_r_^c^* are the numbers of words in the relevant clusters and categories, with *r* labelling the *k* clusters and *c* labelling the categories [23]. Obviously, this is a rather coarse indicator of semantic representation quality, that will depend on the precise clustering algorithm used, but if the vectors for a given set of words do not even cluster according to their known broad semantic categories, there is little hope of them exhibiting appropriate finer grained structure. The correlation of this simple purity measure with performance on the brain activity prediction task will become increasingly clear as this study progresses.

Having defined the main task and performance measures, the remainder of this section presents a systematic series of computational experiments designed to explore the various components of the brain activation prediction task. The first sub-section uses artificially created vectors to explore how the quality of the brain activation vectors (mapping outputs) affect the brain activation prediction results, and the sub-section following that studies the effect of the quality of the semantic representations (mapping inputs). Some further experiments are then presented to clarify the earlier results, and the penultimate sub-section introduces another, even less brain-like, series of artificial brain activation vectors designed to establish what the approach might achieve with more consistent inputs and outputs. The final sub-section considers the relative reliability of the performance measures in the context of the results presented in the earlier sub-sections.

### Artificial Brain Activation Vectors

We have previously shown [18], using the CLUTO Clustering Toolkit [24] with default parameters and cosine distance measure, that the clustering purity of the fMRI brain activation vectors used in this study [8] is low (mean 0.43, standard deviation 0.06, over nine participants). The first aim of the current study is to explore the likely effect of the fMRI vectors' poor semantic representation quality (as indicated by that low clustering purity) on the brain activation prediction task, by generating a series of artificial brain activation vectors covering a range of known clustering qualities and measuring their performance as a function of purity. Of course, generating good semantic representations is difficult [12], [13], [21], even without the requirement for them to mimic patterns of brain activation of varying quality. Consequently, rather than attempting to create a complete representation of semantics on which to base the artificial brain activations, we begin by adopting the simplest possible approach that suffices for current purposes. Later, we shall return to this issue and look at another approach for generating artificial brain activations that leads to a better representation of semantics, at the expense of introducing potential confounding factors.

The simplification that makes this approach feasible is to not attempt to introduce any realistic semantic relations within each category, or between categories, but only require that the categories themselves are clearly defined. Thus the minimal requirement is to have a set of notional voxels for each semantic category whose members tend to have high activation for words within that category, with variation in their activations across different words in that category, and then everything else can be represented by randomly generated activations. That still leaves room for numerous variations, but, fortunately, the general pattern of results does not seem to depend strongly on the details. The simple implementation adopted for the study presented here begins with 4000 artificial voxels in total, each with a baseline activation chosen randomly from the uniform range [0,1]. Then a distinct set of 100 of those voxels is associated with each of the 12 semantic categories, and for each of the 5 words corresponding to each category, a different 80 of the 100 voxels associated with that category have an additional activation *x*. Following the real participants in the Mitchell et al. study [8], nine artificial participants were created, with voxel activation patterns generated for each of the 60 words for each of six “data collection repetitions”, and those activations were normalized, averaged and sorted with respect to stability in exactly the same way as the real data.

**Figure 1:**
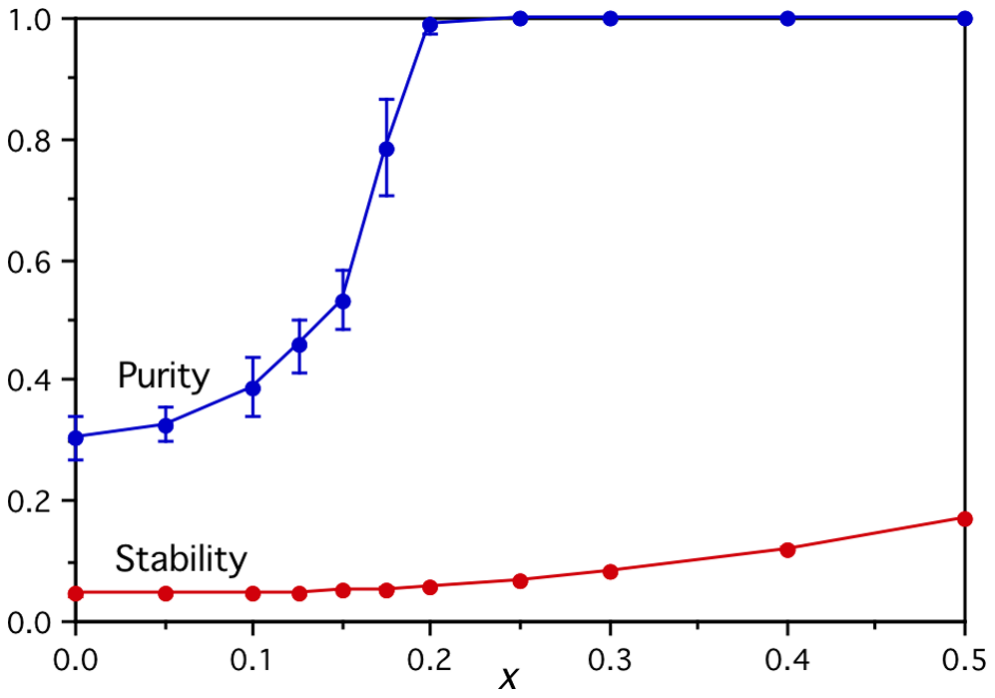
Stability and purity of the artificial brain activations. The stability of the simulated voxel activations over repeated measurements, and their clustering purity based on the associated semantic categories. Both measures increase as a function of the semantic signal level parameter *x*.

The stability of each voxel for each participant is simply defined as the mean correlation of the vectors of activations for the 60 words over all 15 pairs of data collection repetitions [8]. The voxels that have the most stable activations over the six measurements are deemed to provide the most reliable representation of semantics, and it is those that are used in the linear mappings of the prediction task. Figure 1 plots the mean stability and mean clustering purity over the most stable 1000 artificial voxels as a function of the signal parameter *x*. As the value of *x* increases from zero, the semantic signal increasingly stands out from the noise, the stability increases, and the clustering purity increases. Perfect clustering purity is achieved for *x* as low as 0.2, at which point the stability has only reached 0.07. It is reassuring for the whole approach that an effective signal still shows through even with such low voxel stabilities. The stabilities required for good clustering here are rather low compared to the corresponding mean stability of 0.15 for the real fMRI activation vectors [8]. This is probably because the non-signal activations in the six artificial data repetitions are independent of each other, while the real fMRI data will doubtless involve more systematic effects that are unlikely to be adequately approximated by the distribution of random artificial activations. It would be interesting to know the effect of improving the approximations in this respect, as it would be to improve the model in a great many other ways, such as introducing realistic location-specific haemodynamic response functions and other neurobiological constraints. However, the development of such a degree of biological realism would greatly increase the complexity of our models, and require a considerable amount of extra work to justify and validate the biological assumptions and setting of parameters, so that will have to be left for a later study. A related issue is that the real data may well also be subject to a drop-off in quality for later repetitions that we have not attempted to simulate. The effect of both of these simplifications will be elucidated later when we explore how the results depend on the number of data collection repetitions used.

The artificial brain activation vectors can be used in the Mitchell et al. model [8] in exactly the same way as the real fMRI vectors. However, here it proves informative to present the results in more detail. The brain activity prediction task involves a new linear mapping being learned 1770 times corresponding to the 1770 possible pairs of withheld words from the full set of 60 words. Of those pairs, 120 will have both words coming from the same semantic category, and 1650 will have the two words coming from different categories. Here, the results for the 120 harder-to-distinguish within-category pairs (denoted “Within”) and the 1650 easier cross-category withheld word pairs (denoted “Cross”) will be presented separately. The overall performance on the original Mitchell et al. brain activation prediction task [8] is simply the weighted average of those two results.

The models were initially tested using two different sources for the inputs. First, the semantic feature inputs used in the original Mitchell et al. study [8] (and here denoted “M et al.”), based on simple normalized word co-occurrence counts with 25 carefully chosen context verbs derived from the trillion word Google corpus [25]. Second, the improved (and currently best performing) general-purpose semantic representation [12], [21] inputs used in the Levy & Bullinaria study [18] based on word co-occurrence counts derived from the two billion word web-crawled ukWaC corpus [26]. In this case, for each target word *t*, the conditional probability *p*(*c*|*t*) of each context word *c* occurring within in a window of a certain number of words around it is computed. These are then compared with the associated expected probabilities *p*(*c*), that would occur if all the words were distributed randomly in the corpus, to give the Pointwise Mutual Information (PMI) *I*(*c*;*t*) = log(*p*(*c*|*t*)/*p*(*c*)). For low frequency context and/or target words, the observed *p*(*c*|*t*) in the corpus are statistically unreliable, and often become zero leading to negative infinite PMI, which is problematic for most distance measures [27]. Data smoothing or low-frequency cut-off approaches can be used to deal with this issue, but the study of Bullinaria & Levy [12] showed that simply setting all the negative PMI values to zero, leaving vectors of Positive Pointwise Mutual Information (PPMI), reliably resulted in the best performing semantic representations across all the semantic tasks considered, as long as the smallest possible window size (of just one context word to each side of the target) and the standard cosine distance measure were used. Using such vectors based on the 10,000 highest frequency context words generally comes close to optimal for most applications [13], [21], so those are used for the remainder of this study (and here denoted “B&L”).

**Figure 2:**
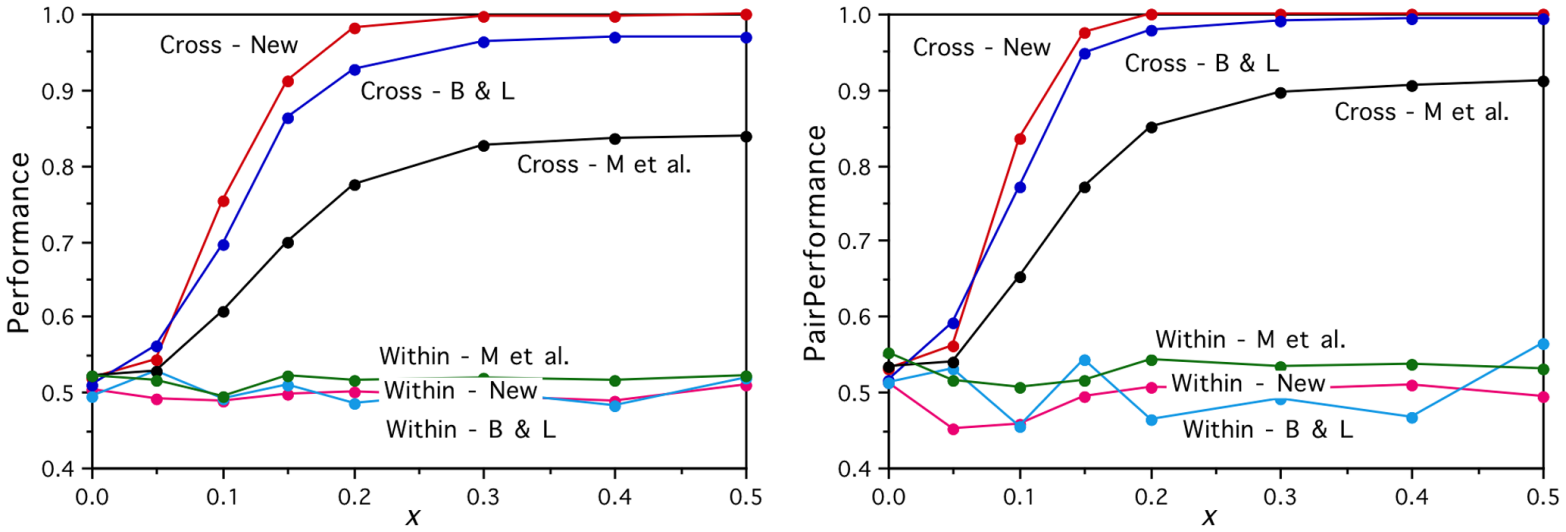
Prediction performance results for the artificial brain activations. For the within-category word pairs (Within), the prediction performances are essentially at chance level as expected. For the cross-category word pairs (Cross), performance increases as a function of the semantic signal level parameter *x,* for all three input semantic feature versions (M et al., B&L, New), and both the *Perf* (left) and *PairPerf* (right) measures.

The graphs in Figure 2 show the results for both feature types using the two performance measures. As expected, the within-category performance is at chance level for all the artificial activations, since the only within-category structure built into them is random, and hence there is no non-random way for the model to choose one word over another in the same category. The cross-category performances all increase with semantic signal *x*, confirming that the models are learning the semantic category structure and the artificial brain activation vectors are performing in the required manner. (The lines denoted “New” are discussed in the next section.)

For comparison, the corresponding models based on the real fMRI activation vectors achieve P*erf* performance of 0.73 (Cross) and 0.57 (Within) using the Mitchell et al. input features [8], and 0.78 (Cross) and 0.55 (Within) using B&L input vectors [18], and the corresponding *PairPerf* results are 0.81, 0.60, 0.86 and 0.62 respectively. So, the cross-category model performances using the real brain activations are worse than those using the artificial activation outputs with semantic signals *x*≥0.15, which indicates that the quality of the measured brain activations is at least one of the serious limiting factors for the linear mapping approach.

**Figure 3:**
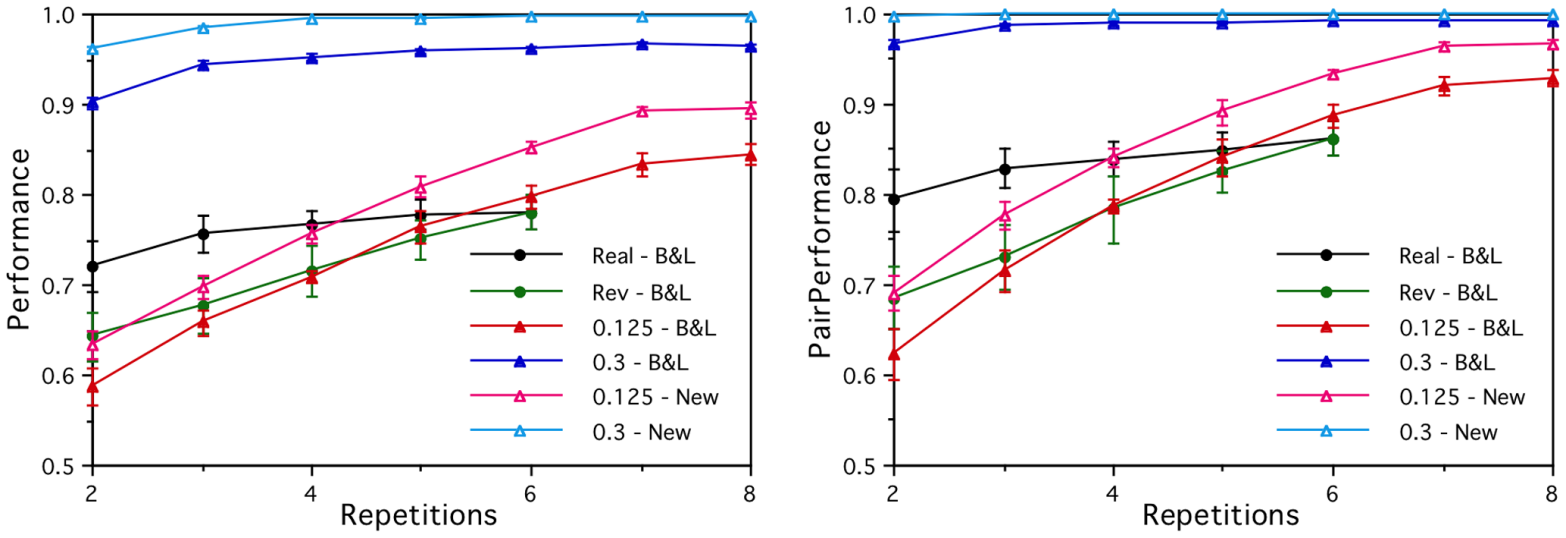
Dependence of the prediction performance results on the number of data collection repetitions. There is a general increase and then levelling off of performance with number of repetitions for each of the real (Real), reversed real (Rev), and artificial (*x* = 0.125, 0.3) brain activations for cross-category word pairs, for both input semantic feature sets (B&L, New), and both the *Perf* (left) and *PairPerf* (right) measures.

**Figure 4:**
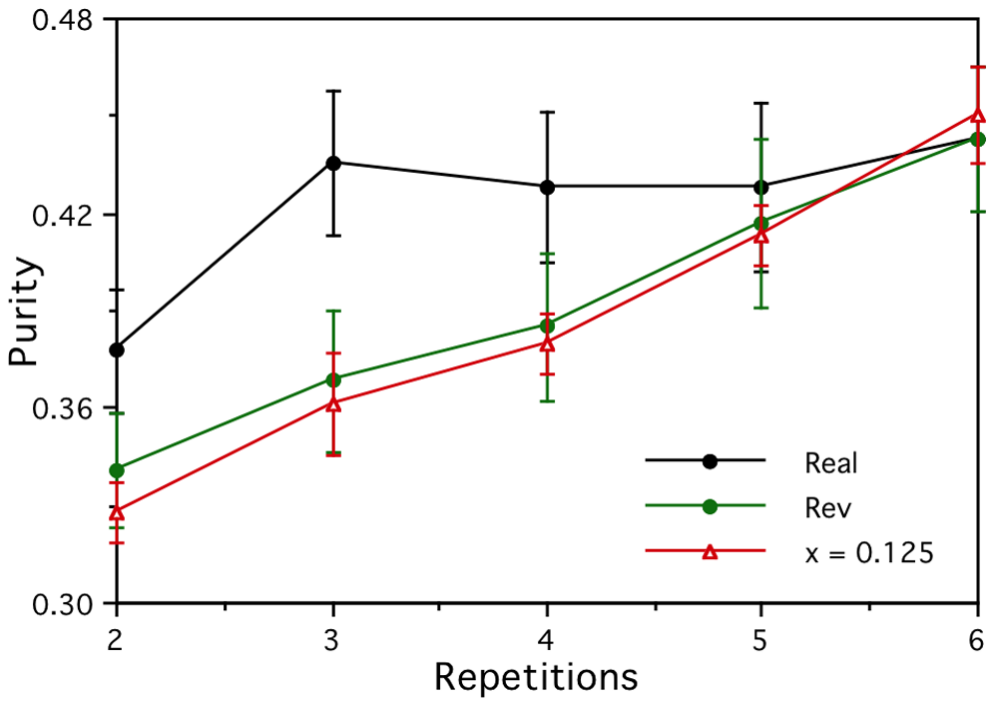
Dependence of the clustering purity on the number of data collection repetitions. The clustering purities of the real (Real), reversed real (Rev) and artificial (*x* = 0.125) brain activations all follow similar patterns to the corresponding brain activity prediction performances seen in Figure 3.

It is natural to ask what might be done to improve the real brain activation vectors. From a noisy data collection perspective, one would expect a cleaner signal to emerge by averaging and determining stability over more measurements for each word. Figure 3 shows that for the artificial brain activation vectors with *x* = 0.125 and B&L semantic vectors there is steady improvement in performance on the prediction task from two data measurements (the minimum required to measure stability) up to seven, but then there is a ceiling effect levelling off. For the corresponding *x* = 0.3 stronger signal case, the performance reaches ceiling levels after only three or four data measurements. For the real brain activations, there is again little increase in performance to be gained by using more than the first three or four measurements out of the six collected, but it is not obvious why. In fact, the graphs show that reversing the real data (i.e., using the last sets of measurements rather than the first) results in the performance deteriorating much more rapidly as the number of measurements is reduced from the full set of six and fewer of the early measurements are used. This is presumably because of the demands on the participants as they lay in the scanner for more than an hour generating brain activations for six repetitions of the word set in a single continuous run. Clearly, more data collection repetitions allow a better signal to emerge from the noise, as shown with the artificial brain activations for which the order of the measurements makes no difference, but that is limited by how much useful signal there is to be found. The longer the participants are in the scanner, the more they will tend to move, and the more poorly they are likely to perform due to fatigue. That will lead to more noise in the later repetitions and less to be gained by using them. This pattern is equally clear in the dependence of the simple clustering purities on the number of data collection repetitions shown in Figure 4, which provides further evidence that the purities are a useful indicator of the performance that can be expected on the harder prediction task.

### Improved Corpus-Based Semantic Vectors

It is evident from Figures 2 and 3 that, even when the models' outputs are the perfectly clustering artificial activations with *x*≥0.3, the performances are still not perfect. All these results inevitably also depend on the quality of the semantic representations used for the models' inputs. The clustering purity of the Mitchell et al. semantic feature inputs [8] is 0.47, and for the B&L input semantic representation it is 0.83, and that difference is clearly reflected in the models' performances seen in Figure 2. Consequently, it is natural to ask whether improved semantic representation inputs with perfect clustering could result in better models. Murphy, Talukdar & Mitchell [19] have already compared a number of alternative corpus-based representations as inputs for the brain activation prediction task, but none of them perform any better than the B&L vectors we have been using.

Unfortunately, the Mitchell et al. word set [8] contains several problematic words that render it impossible to obtain perfectly clustering semantic vectors using standard corpus co-occurrence statistics based approaches [12], [13], [20], [28], so we first need to optimise the word set for this kind of semantic representation. The main problem is that words which have multiple meanings will result in combined semantic vectors that match no single meaning and therefore cluster poorly. Another issue is that words in diverse categories, and single words that are outliers in (or unusual members of) their semantic category, also tend to cluster poorly. Dealing effectively with such words is not straightforward (e.g., [28], [29]), and this matter will clearly need to be addressed in the future, but for the current study, that aims to see how well the existing brain activation vectors could perform given more reliable semantic vectors, we can proceed by simply avoiding the problematic words. Fortunately, the CLUTO Clustering Toolkit [24], that is already being used to determine the clustering purities, also allows the word clustering to be plotted as dendrograms in which any problematic words can be easily identified for replacement [21]. The simplest way to improve the Mitchell et al. word set [8] was found in that way to be by replacing two problematic categories (*man made objects*, too diverse, replaced by *fruit*; *furniture*, too diverse and ambiguous, replaced by *birds*) and five other problematic words (*bear*, not always the *animal*, replaced by *pig*; *saw*, not always the *tool*, replaced by *spanner*; *glass*, not always the *kitchen utensil*, replaced by *bowl*; *knife*, not always used as a *kitchen utensil*, replaced by *plate*; *igloo*, class outlier, replaced by *cottage*). These changes prove to be sufficient to result in an improved word set (denoted “New”) that has B&L style semantic vectors [12], [21] which cluster perfectly (i.e., with purity of 1.0).

The graphs in Figure 2 show the artificial brain activity prediction task performance with those improved input vectors (New) on the within- and cross-category withheld word pairs. Now, perfect performances on the cross-category task are achieved using the perfectly clustering artificial activations with *x*≥0.3, and the within-category task performances remain at chance level as expected for all values of *x*. Similarly, Figure 3 shows that the improved input vectors result in significantly enhanced cross-category performance for all numbers of data collection repetitions. This establishes the importance of having a good test word set, for which a good semantic representation is possible, and confirms that the linear mapping approach is able to perform perfectly on the cross-category prediction task given perfectly clustering inputs and outputs. Later, an alternative series of artificial brain activation vectors will be developed that allows us to test the limits of the linear mapping approach on the harder within-category prediction task too.

**Figure 5:**
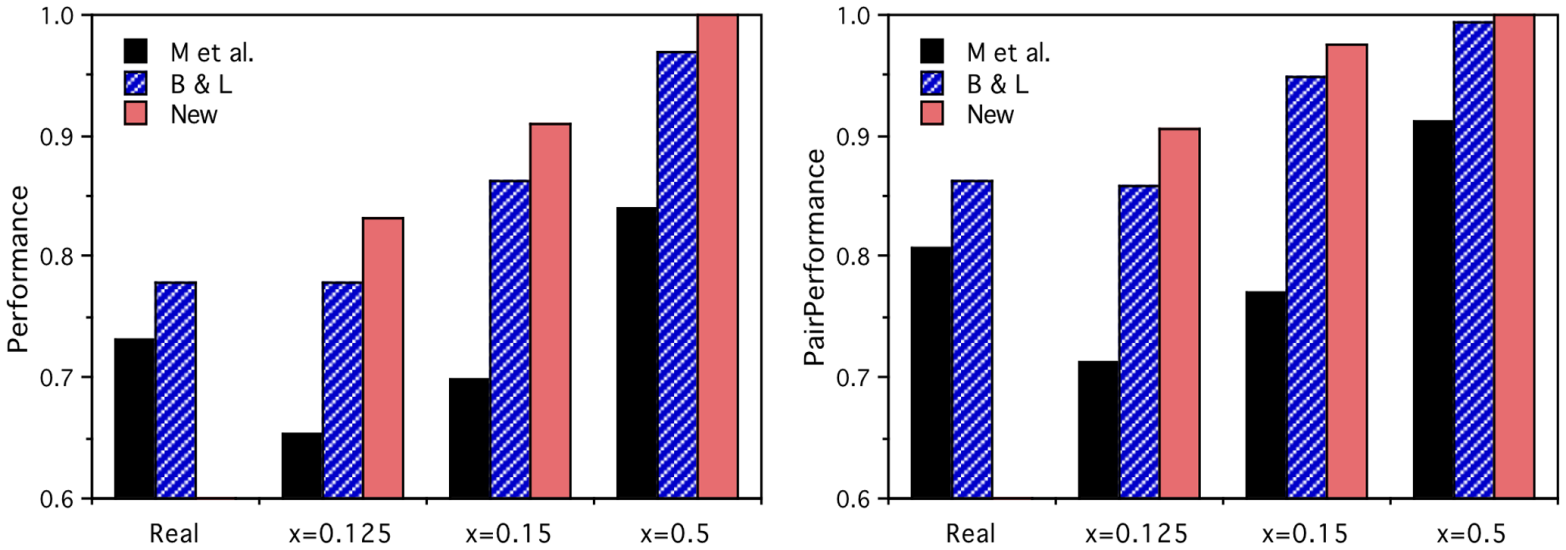
Comparative brain activation prediction performance results. Performance of the real (Real) and artificial (*x* = 0.125, 0.15, 0.5) brain activations for cross-category word pairs, for the three input semantic feature versions (M et al., B&L, New), and both *Perf* (left) and *PairPerf* (right) measures. These comparisons provide the first indication that it is the quality of the brain activation data that is the main factor limiting performance.

Since we have no fMRI data for the new words in the improved word set (New), they cannot be tested on the prediction task using real brain activation outputs. However, the clustering purity of the artificial activations with *x* = 0.125 matches the clustering purity of the real fMRI vectors, so those artificial activations might provide an indication of how well the real activations would perform with the improved word set. The prediction performances of the artificial activations for that and selected higher values of *x* are plotted in the histograms of Figure 5 for the three input vector types, with the corresponding results for the real brain activation vectors (Real) with the two input vector types for the original word set. It is clear that the improved word set on its own only provides rather limited, albeit significant, prediction task enhancement. Interestingly, there is a close correspondence between the *x* = 0.125 and real activation prediction results for the B&L input features, but the Mitchell et al. input features [8] produce much better results with the real activations than the artificial activations would suggest. The reasons for that are certainly worthy of further exploration.

Another way to explore the effect of better semantic inputs on models using real fMRI outputs would be to look for subsets of the original 60 Mitchell et al. words [8] that cluster better, and see how well they perform with the real fMRI vectors. This can be done by removing the *furniture*, *man made objects*, and *kitchen utensil* categories from the full set of 60 words to leave nine categories of five items that lead to B&L style semantic vectors [12], [21] which cluster perfectly (with purity of 1.0). Obviously, the prediction task performance will fall with the number of training items [18], but these good 45 words can be compared with the corresponding results obtained using random sets of 9 categories and random sets of 45 words. On the main Mitchell et al. prediction task [8] with real brain activation outputs, this results in average *Perf* performances of 0.765, 0.739 and 0.743 respectively, and corresponding average *PairPerf* performances of 0.844, 0.819 and 0.827. The differences are small, but paired *t* tests on the nine participants' results show that the semantic vectors for the chosen good words perform significantly (*t*(8)>3.36, *p*<0.01) better than those of both random sets, using either performance measure, again confirming the importance of having good semantic vector inputs for the models.

Finally, it is possible to get an idea of the contribution of the quality of the real brain activation vectors to this less-than-perfect performance on the 45 word subsets by running the same tests using good quality (*x* = 0.5) artificial brain activation vectors. In this case, we obtain *Perf* performances of 0.954, 0.924, 0.923 and *PairPerf* performances of 0.958, 0.950, 0.960, and the chosen good words do not perform significantly differently from the expected ceiling of 0.955 (derived assuming chance performance on the within-category pairs). These results indicate that, while having more reliable B&L semantic vector inputs does enable improved prediction performance, it is the quality of the brain activation vectors that remains the main limiting factor.

### Further Measures of Performance

One crucial difference between real brain activation vectors and the artificial version discussed above is that the artificial activations only represent the distinctions between the 12 Mitchell et al. [8] semantic categories, and it would obviously be better if they could include finer grained structure that allowed more realistic semantic relations within and between those 12 high-level categories. Unfortunately, those finer grained semantic relations are enormously complex, and building them into the artificial vectors by hand would be a huge task, even if we had already solved the difficult task of establishing what form they should take. In practice, that is not really feasible, even for the relatively small sets of 60 words used here. What we can and should do, however, is explore the consequences of that simplification.

Since the category labels of the simple artificial activations discussed above are assigned randomly, and all the categories have an equivalent randomly generated form, they can clearly be swapped around with no change to the resulting performances. Similarly, if the members within each category are swapped around, the performances do not change. The complete lack of cross-category semantic structure can be confirmed by using one of Mitchell et al.'s supplementary tasks, that was designed to see how much the performance dropped if, for each pair of withheld words, all the other words from their respective categories were withheld from training too [8]. For the real fMRI vectors, the *Perf* performances drops from 0.721 and 0.763 (for the Mitchell et al. and B&L input features) down to 0.668 and 0.664, and the *PairPerf* performances drop from 0.793 and 0.846 down to 0.736 and 0.793, but they all remain significantly better than chance. The same measures for the artificial brain activation vectors all drop to chance levels, as expected. The implication is that the real brain activation vectors contain a lot more useful information than simply the highest level categories, and any pre-processing of them that leads to improved clustering at the expense of the finer grained structure will render them closer to the artificial vectors and lead to similar limitations.

Although both the original Mitchell et al. [8] and improved B&L [12], [21] semantic feature vectors are derived from large text corpora, they are generated by different computational processes, have massively different dimensionality (25 and 10,000), and inevitably have rather different internal structures. It is natural, therefore, to ask how much their fine-grained structure, beyond the clustering into 12 broad categories, contributes to the performances on the brain activity prediction task. Randomly reassigning the feature vectors to the wrong words would clearly cause the performance to drop to chance levels, because that destroys all the semantic structure. However, it is not obvious how the results would be affected if the feature vectors were randomly reassigned in a way that preserved the main category structure, i.e. all the words within one category were only assigned feature vectors that really corresponded to words from within a single other category. To test that, one can take the perfectly clustering feature vectors (New), randomly swap the categories with the original word set in such a way that none are correct, and retrain the models. That obviously makes no difference to the perfect cross-category and chance within-category performance for the artificial activation vectors, because all the categories there are equivalent. For the real fMRI activation vectors, the resulting cross-category performances are 0.73 for *Perf* and 0.80 for *PairPerf*, which are significantly worse than the corresponding results of 0.77 of 0.86 for the genuine B&L feature vectors, but they are still highly statistically significantly better than chance. Interestingly, there is no significant difference between these category randomized B&L feature results and the genuine Mitchell et al. feature results (0.73 and 0.81). Naturally, since the within-category semantic structure is now essentially random, all the within-category performances have dropped to chance level.

One can take this idea even further and use randomly generated “semantic features” that have the main category structure and see how well they perform. Obviously there are lots of ways that could be done, but one simple approach will suffice to illustrate what typically happens. Twelve random 25-dimensional vectors were created with components drawn uniformly from the range [0, 1] to represent 12 category centres, and then to each of these were added five different random perturbation vectors with components drawn uniformly from the range [0, 0.2] to give 60 feature vectors. Only about one in four of the resulting vector sets clustered with perfect purity, but after 35 attempts, ten sets of random vectors with the required category structure were obtained. These were then each used as inputs in the main brain activity prediction task using real fMRI activation vector outputs as described above, and the average results over the ten random sets computed. Obviously, the within-category performances were again at chance level, because the within-category structure of the inputs is random, but the cross-category performances were 0.74 for *Perf* and 0.81 for *PairPerf*, which are again significantly worse than the corresponding results of 0.77 of 0.86 for the B&L input vectors, but still significantly better than chance, and not significantly different to the results of 0.73 and 0.81 for the Mitchell et al. input features [8]. The implication is that surprisingly good statistically significant results can be achieved with any input vectors that have the right high-level category structure, irrespective of whether they correspond to a semantic representation based on real empirical linguistic measurements.

**Figure 6:**
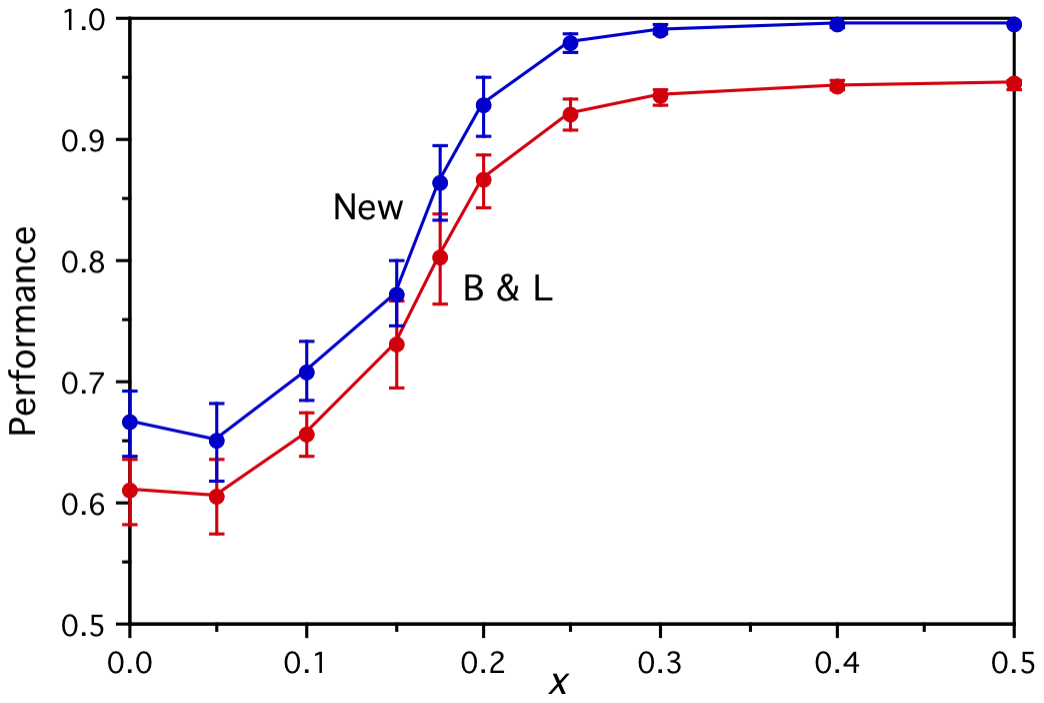
Single word prediction performance. Ranking the similarity of the actual brain activations to the predicted activations for each test word and 1000 control words provides an alternative measure of performance as a function of the artificial brain activations' semantic signal level *x*, for the two input feature sets (B&L, New). This exhibits the same general pattern of prediction results as seen for the main task in Figure 2.

The remaining supplementary task used by Mitchell et al. [8] was designed to investigate how well the brain activation prediction models perform when faced with large numbers of inputs not from their 60 word set. For each of 1000 control words (selected due to their ranking 301 to 1300 in frequency in the corpus), corpus-derived semantic vector inputs were created as before. These were then passed through each of the 60 models generated by training on 59 of the 60 words, and for each model the similarity of the withheld word brain activation pattern with each of the 1000 control word outputs and 1 withheld word output were ranked. The higher the withheld word ranks on average, measured as a fraction of the other 1000 words falling below it, the better the models' prediction performance. The measured performances using the improved input semantic vectors (B&L) and the corresponding improved word set vectors (New) with the artificial brain activation outputs of varying quality are shown in Figure 6. Both input types show increased performance as a function of *x*, reaching ceiling levels around *x* = 0.4. The New (perfect purity) vectors perform significantly better than the original B&L vectors (reaching 0.995 rather than 0.945), again confirming the advantage of using a word set that allows good semantic features. (Note that perfect performance is not expected here, even with perfect features and perfect artificial activation vectors, because the 1000 words include some closely semantically related words that are effectively within-category and the artificial activation vectors therefore have no way to distinguish them.) By comparison, using the real Mitchell et al. fMRI activation outputs [8], the B&L input features achieve a performance on this task of only 0.81. Once again, this indicates that it is the brain activation vectors that are the main limiting factor of the mapping approach.

### Corpus-Based Artificial Brain Activations

As noted above, the artificial brain activation vectors might lead to more informative results, with better than chance level within-category performances, if they had a more refined structure than merely the highest level semantic categories. However, the best semantic representations currently available are the corpus-based representations that are already being used as the inputs to the models [12], [21], and using those as a basis for modelling the outputs as well would clearly not be very realistic since it would reduce the brain activity prediction task to simply learning an identity mapping. Moreover, if such vectors were taken to be the underlying representation and six versions of added noise were combined with them to simulate the six repetitions of the fMRI measurements, the simulated voxels selected by their stability would be a relatively small random subset of the full set of 10,000 corpus vector components, and they would not perform well [12]. It is possible to take this idea a little further, though, because there exists a transformation of the standard B&L corpus-based semantic representation that uses a weighted version of Singular Valued Decomposition (SVD) to reduce the dimensionality of the vectors and flatten the relative contribution of the remaining vector components [13], [30]. That transformation leads to semantic representations with significantly improved performance on some semantic tasks [13], but has a relatively modest effect on the brain activity prediction task of interest here. However, it is useful in that the transformed semantic representation can be taken to form the basis of another series of artificial brain activation vectors. Obviously, they will be rather unrealistic as a model of real brain activation vectors, but they may, nevertheless, provide a useful approach for estimating how much real training data might be required to learn the prediction task mapping. The idea is that if the simplified mapping based on these artificial vectors cannot be learned with a certain number of words, it is unlikely that the real mapping with real brain activations will either.

These artificial brain activation vectors are not a simple transformation of the 10,000 component B&L vectors used as the models' inputs for the 60 target words. Rather, one starts with the matrix *M* of B&L style corpus-derived semantic vectors consisting of 50,509 component vectors for each of the 50,548 highest frequency target words, and SVD allows the original matrix to be written in the form *M* = *USV* ^T^, where *U* and *V* are orthogonal matrices, and *S* is a diagonal matrix containing the singular values in decreasing order. (The precise size of the starting matrix *M* is not crucial – larger matrices do not improve what emerges, though much smaller matrices can lead to worse performance [13].) Then the vectors *Y* = *MV* = *US* are principal components that can be truncated at an optimal number of dimensions, and can also be scaled by positive or negative powers of the singular values to allow emphasized contributions from the earlier or later components [30]. That scaling can be optimized for the chosen application (in this case, by maximizing performance on independent validation tasks that also require good semantic representations) leading to the vectors *X* = *US* ^0.25^ which are weighted principal components that prove to be equally good or better semantic representations than the original vectors *M* [13]. These can then be used to generate artificial brain activations by starting with vectors that are the first 1000 dimensions of *X* for our chosen word sets, creating six different noisy versions by adding random noise drawn uniformly from the range [−*z*, *z*] to represent the six repetitions of the simulated fMRI measurements, and again using the same normalization, averaging and sorting with respect to stability as with the real data. This gives a new series of artificial brain activation vectors parameterized by the noise value *z*. These allow us to simulate the (probably unachievable in practice) limiting case in which the semantic representation input for the brain prediction task has a simple noisy linear relation to the brain activations. If the brain activation prediction models cannot perform well in this case, then they probably never can.

**Figure 7:**
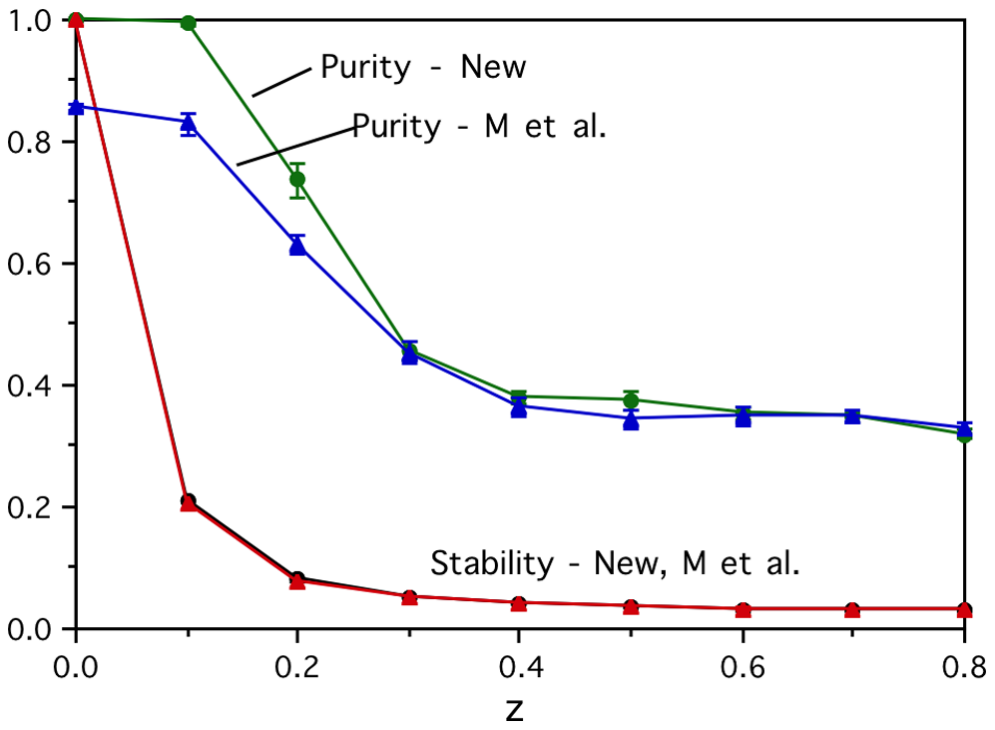
Stability and purity of the corpus-based artificial brain activations. The stability of the artificial voxel activations over repeated measurements and their semantic clustering purity both fall as a function of the noise parameter *z*, for both the original word set (M et al.) and the improved word set (New).

**Figure 8:**
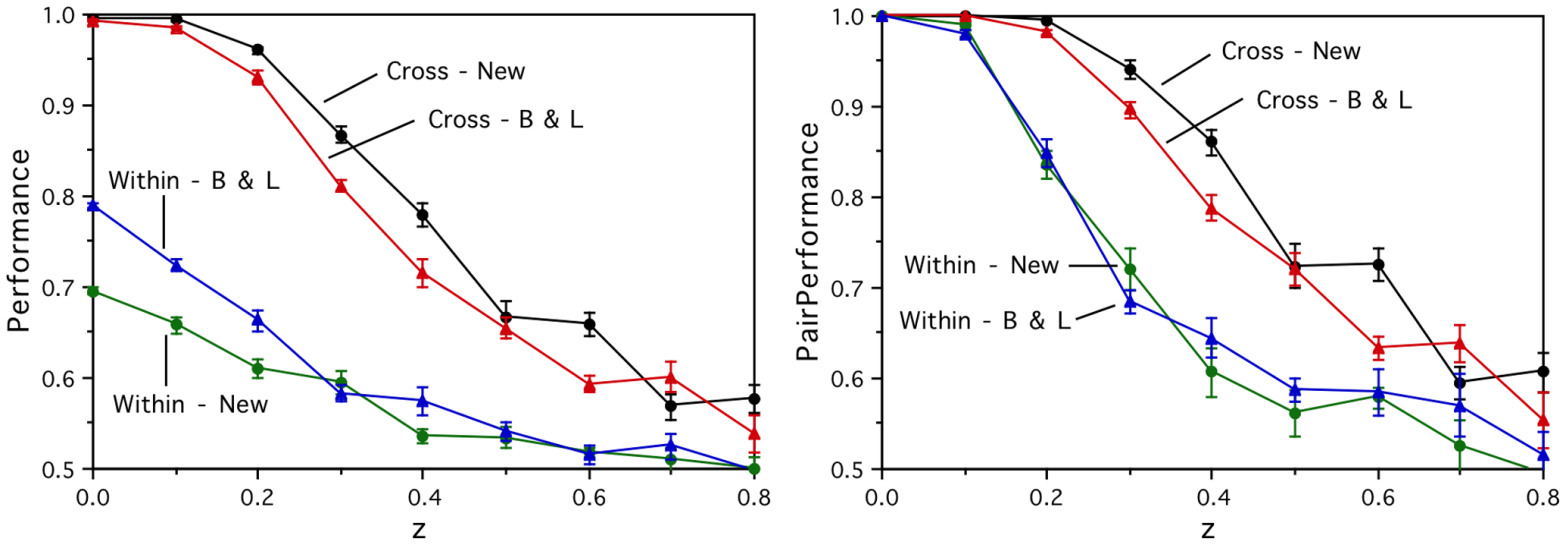
Prediction performance results for the corpus-based artificial brain activations. All the performances fall as a function of the noise parameter *z*, for within- and cross-category word pairs (Within, Cross), two input semantic feature sets (B&L, New), and both *Perf* (left) and *PairPerf* (right) measure.

Figure 7 shows how the stability of these new corpus-based artificial brain activations fall with the noise parameter *z*, independently of which word set is used. It also shows how their semantic clustering purity falls from the noise free levels (of 1.00 for the improved word set denoted “New”, and 0.86 for the Mitchell et al. word set denoted “M et al.”) to a floor of about 0.35. The corresponding falls in performance on the brain activation prediction task using B&L style input semantic vectors are shown in Figure 8 for the original word set (denoted “B&L”) and the improved word set (denoted “New”). The cross-category performances (both *Perf* and *PairPerf*) are now near perfect for both word sets for zero noise *z*, but the improved word set performances fall more slowly for moderate noise levels. For a noise level of *z* ∼ 0.35, all the brain activity prediction performances are in reasonable agreement with those arising from using the real fMRI data in the same way.

The more realistic fine-grained semantic structure of these corpus-based artificial brain activations leads to within-category performances that are now significantly better than chance, but they are still far from perfect. Here the poorer quality M et al. word set performs better (*Perf* of 0.79 rather than 0.70, for the zero noise case), as expected given that many of its “within-category” vectors are not really within their nominal category. The relatively poor within-category performance (*Perf*) on the New word set indicates that 60 words are insufficient to provide enough information for the linear models to learn fine-grained semantic distinctions from the corpus-based representations.

**Figure 9:**
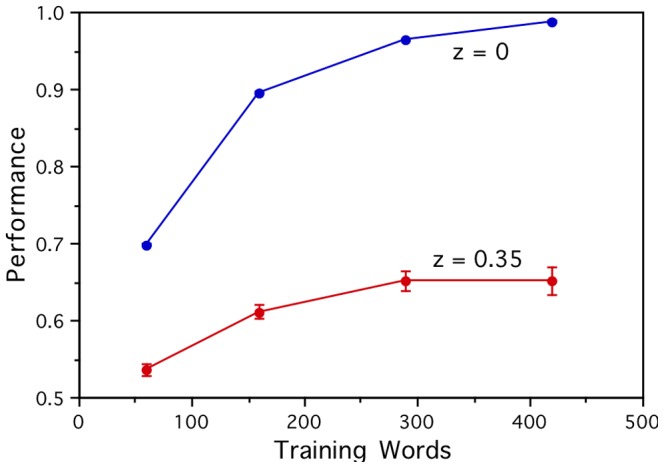
Dependence of the within-category prediction performance on the number of training words. Prediction results on the New word set for noise free (*z* = 0) and noisy (*z* = 0.35) corpus-based artificial brain activations, as a function of the number of words used to train the linear models. These results indicate that many more than the 60 words currently used will be required to achieve good performance, even for much cleaner brain activation data.

Establishing how many training words are required for the whole approach to work well is obviously important, particularly given the difficulties involved in obtaining good quality fMRI measurements for large numbers of words. One advantage of working with artificial brain activations is that it is relatively easy to generate them, and that makes it feasible to explore how the performance improves as more words (in addition to the 60 word test set) are used to train the linear models. This was done using 100, 230 and 360 additional words from the *Distance Comparison* test set of Bullinaria & Levy [12], [13]. Figure 9 shows how the within-category performance (*Perf*) for the New word set improves as a function of the total number of training words. For the noise free case (*z* = 0), the performance quickly improves from the 60 word level seen in Figure 8 to near perfect performance. The noisy case (*z* = 0.35) also shows improvement, but reaches a relatively low ceiling of 0.65 by about 300 training words. So, more training words do help, but that alone is not likely to be sufficient to overcome the current noise levels in the brain activation vectors.

### Reliability of the Performance Measures

Throughout this paper, we have taken the trouble to present the results using both the *PairPerf* measure used in the earlier studies [8], [14]–[20], and the *Perf* measure [18] that we consider to be more useful in practice, because it provides an estimate of the probability that the model predicts the right output rather than a given random alternative. In most cases, the *Perf* and *PairPerf* results have followed the same pattern, with *Perf* taking on slightly lower values. However, for the zero noise case in Figure 8, both the within and cross-category paired performances are perfect, even though the *Perf* graph shows that up to 30% of the individual within-category predictions are actually wrong. Clearly, this discrepancy could give a misleading impression of the performance of the model, and it is consequently important to investigate further what is underlying it.

**Figure 10:**
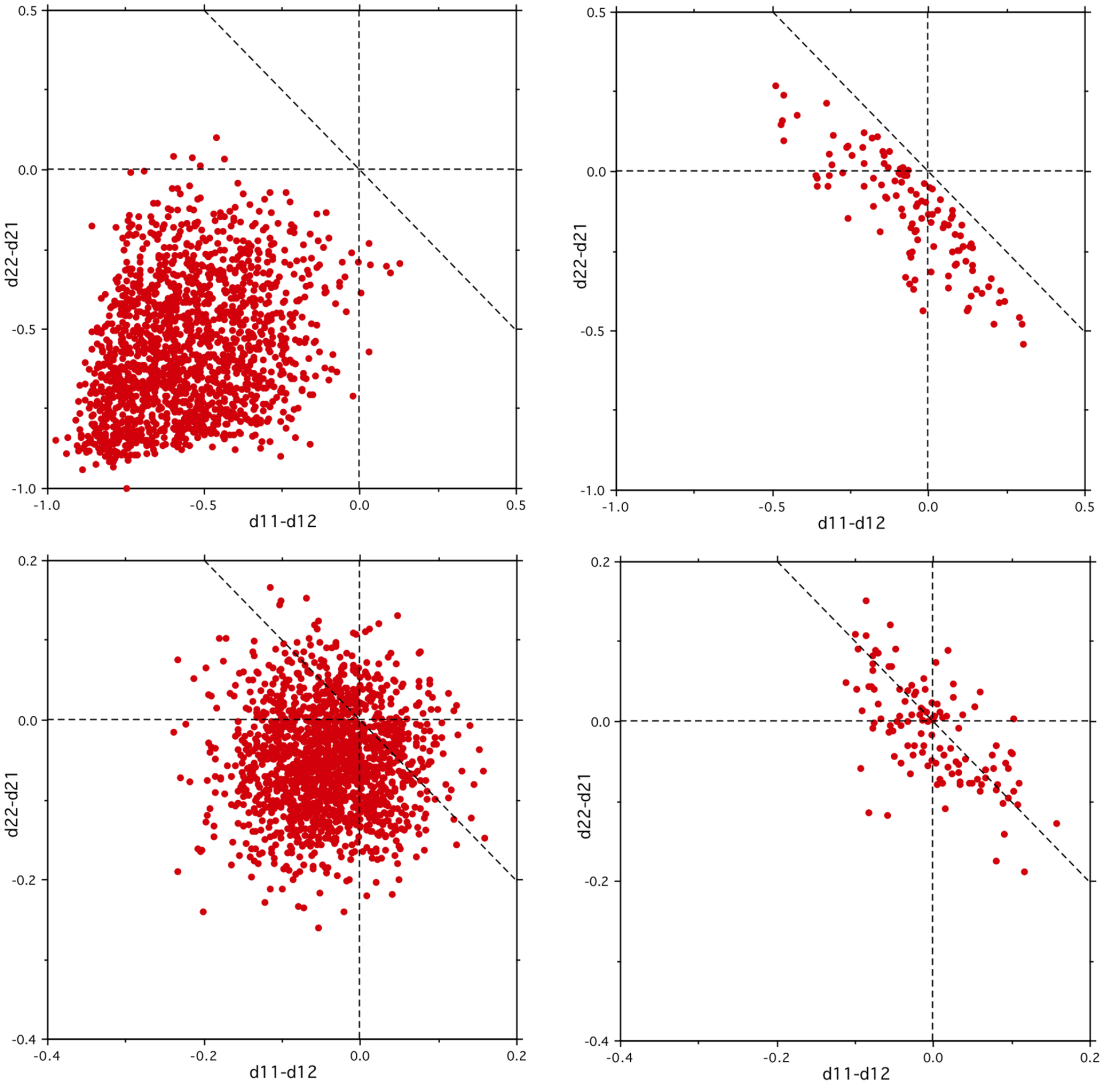
Individual distance differences underlying the measures of performance. The results from Figure 8 are shown for *z* = 0.0 (upper), *z* = 0.4 (lower), cross-category (left) and within-category (right). The *Perf* measure is the percentage of data points with distance differences (*d11*−*d12* and *d22*−*d21*) that are less than zero. The *PairPerf* measure is the percentage of data points with combined distance difference (*d11+d22*−*d12*−*d21*) less than zero, i.e. below the diagonal dotted lines in the graphs.

Both measures are based on the cosine distance between the model output and the corresponding actual brain activation (distance *d11* for input word 1 and *d22* for input word 2) and the cosine distance between the model output and the brain activation for the other word (*d12* for input word 1 and *d21* for input word 2). A correct prediction for word 1 has *d11*<*d12*, and a correct prediction for word 2 has *d22*<*d21*. *Perf* is simply the percentage of correct predictions, while *PairPerf* is the percentage for which *d11*+*d22* < *d12*+*d21*. To explain how a big discrepancy between the two measures can arise, Figure 10 plots the crucial distance differences for the cross- and within-category pairs, for the New dataset, corresponding to one *z* = 0.0 and one *z* = 0.4 simulated participant used to generate the results of Figure 8. For the cross-category case with no noise (top-left graph), all the correct-word distances (*d11* and *d22*) tend to be much less than 1 and the wrong-word distances (*d12* and *d21*) near 1, so there are few prediction errors, and none for which both the pair of words is wrong. When noise is added (bottom-left graph) all the distances are around 1, there are many more prediction errors, and correspondingly more *PairPerf* errors. One might expect the harder within-category task for zero noise (top-right graph) to follow a similar distribution to the high noise cross-category case, but that does not happen. Instead, the nature of the mapping means that the two components of the paired measure are not independent, but anti-correlated with the correct result dominating. Even though there are many individual prediction errors, the paired measure does not show any. There is still a noticeable anti-correlation in the high-noise within-category case (bottom-right graph), but the effect on the *PairPerf* measure is not so dramatic there.

Since the standard prediction task [8] is dominated by the cross-category word pairs, and the real brain activation data is very noisy, this potentially misleading aspect of the *PairPerf* measure will not have made much difference to the patterns of results presented in previous studies, but the results for the artificial brain activations in this paper lead us to suggest that using the *Pair* measure would be a more reliable approach for future studies of this type.

## Conclusions and Discussion

In view of the considerable recent interest in the idea that linear mappings from general-purpose semantic representations to patterns of fMRI brain activity could be a fruitful avenue for helping to understand the representation of semantics (or lexical/conceptual meanings) in the human brain [8], [14]–[20], this paper has explored the key factors which currently limit that approach. Studying improved corpus-based semantic representations and two parameterized series of artificial brain activation vectors has led to the conclusion that better brain activation prediction performance *is* achievable with better semantic feature input vectors *or* better brain activation vectors, but the improved B&L semantic vectors [12], [21] are already close to ceiling quality for non-ambiguous concrete nouns. We have also shown that surprisingly good performance can even be achieved with input feature vectors that do not correspond to the right words at all, as long as they have the right high-level semantic category structure, so one has to be careful when drawing conclusions simply because the performance levels are statistically significantly better than chance.

It has become clear how the brain activation prediction models' ability to distinguish words within the same semantic category is a more challenging sub-task, and that may provide a more reliable indication of the limits of the whole approach. Of course, it is not surprising that the linear mapping approach is better able to distinguish between semantically unrelated words than it is between words within the same semantic category, particularly for the relatively small word sets used so far. The empirical results presented in this paper indicate that, with cleaner brain activation vectors, the approach should be capable of working well on non-ambiguous concrete nouns for the easier cross-category task, but it remains to be seen how well it will be able to perform on the within-category task, or how technically feasible it will be to obtain better fMRI vectors. The results from studying corpus-based artificial brain activations suggest that larger word sets for training the mapping will be required to distinguish nouns with closely related semantics. For other word types, such as verbs and homographs, there are known problems with generating good corpus-based semantic representations for use as the input features [12], [20], [28], so it remains unclear how well the approach will ever be able to work for them. However, recent work on this matter (e.g., [19], [29]) suggests that further progress should be achievable.

Taken together, the experimental results presented in this paper strongly suggests that it is the lack of representational distinctiveness of the fMRI voxel activation vectors that is the major limiting factor to further improvements in the Mitchell et al. style learning models [8], [14]–[20]. There is compelling evidence that the brain activation vectors *do* contain significant categorical and item-based semantic information, but the linear models fail to generalize at anything near the level of the human ability to categorise and identify individual items. The results of Figure 3 suggest that simply collecting more data for each test word with the Mitchell et al. approach [8] has already reached a performance ceiling. It may be the case that fMRI technology is never going to be able to measure semantic representations in the brain at an appropriate “grain size”, either due to the lack of sufficient field strength or other technical limitations, or due to the vascular source of the Blood Oxygen Level Dependent (BOLD) signal not reflecting neural representations precisely enough. However, this pessimism may be premature, since further experimental paradigms for data collection have yet to be explored. These will certainly include a range of different semantic domains and experimental designs. For example, Wang, Baucom & Shinkareva [31] have already investigated an experimental paradigm that should lead to less general and diffuse brain activation than the property generation approach of Mitchell et al. [8], and demonstrated in a decoding task that single-trial brain activation vectors can reliably distinguish between concrete and abstract words at above chance levels, though performance on distinguishing individual words remains rather low. Moreover, we have shown in the current study that there tends to be a fall off in quality for later fMRI data collection repetitions, and our results from corpus-based artificial brain activations suggest that the datasets may need to involve considerably more than 60 words to provide good results, so future experimental paradigms may need to use event-related designs over multiple runs, and even multiple sessions, in order to collect enough good quality data. Raizada & Connolly [32] go further and suggest neural activation decoding across subjects purely within neural similarity space.

The general way forward for the Mitchell et al. [8] style brain activation prediction task seems to be clear: choose word sets for which high quality semantic representations are possible, and then try to identify ways of obtaining brain activation vectors that perform better. Choosing word sets with good semantic representations appears straightforward using the corpus-based approach of Bullinaria & Levy [12], [21], and there is plenty of scope for accommodating more sophisticated hierarchical semantic structures that will allow finer-grained investigations than the simple high-level categories used so far. The stimuli used by Mitchell et al. [8] were pairs of concrete nouns and simple line drawings of the concepts denoted by those nouns. Just et al. [22] and Shinkareva et al. [33] have demonstrated that similar results can be obtained using purely lexical stimuli. In both cases, visual cortical areas are included in the set of most stable voxels, and it is possible that the properties of the stimuli, along with the property generation task used in the experimental paradigm, encourage more purely visual representations than other tasks that might be more purely conceptual. It would be interesting to explore whether auditory presentations of word stimuli, or experimental tasks that are passive (e.g., [34]), or demand judgements of semantic similarity (e.g., [35]), produce different results in models similar to those of Mitchell et al.

There are also several ways in which the fMRI data could be collected and/or pre-processed differently, that might better capture the voxel activation patterns underlying the important semantic distinctions in future data-sets. The fMRI data collected by Mitchell et al. [8] are in the form of rather large voxels (3.125×3.125×5 mm, with a 1 mm gap between slices, re-sampled to 3×3×6 mm) measured over brief (1 second) scans. The fMRI signal depends on blood flow, and this is relatively slow compared to the dynamics of cognitive processing. Mitchell et al. took this into account by discarding the first three scans and taking the mean of the next four. This is a rough approximation of the usual fMRI pre-processing step of convolving the data time series with a continuous canonical haemodynamic response function. Their approach produced fMRI data that demonstrated the feasibility of the modelling approach, and was easily re-analyzed when released to the research community. However, it is possible that different details might lead to improved performance on the prediction task. Longer scan times of around two or three seconds could allow the sampling of smaller voxels, and that might enable better performance, though changes in the timing parameters may necessitate changes in the task required of the participants. The merits demonstrated in this paper for collecting data on greater numbers of words or concepts suggest that future experiments may have to be broken up into multiple fMRI runs and sessions anyway. That will clearly pose additional data processing challenges, but could mitigate some of the current problems with the introduction of noise in long runs due to fatigue and head movements. It is not obvious whether any of these scanning or pre-processing changes will really be able to improve the data sufficiently, but this might prove to be the best way for future research in this area to make advances.

Another possibility remaining is that more complex variations on the linear models, or more sophisticated learning and regularization approaches, may be able to perform better with the existing fMRI data, for example, by extracting more of the signal that is potentially still hidden in that data. Some interesting experiments with different regularization methods and multi-task learning have already been proposed by Liu, Palatucci & Zhang [36] and Chen et al. [37], though no techniques have yet been found to work much better on the original Mitchell et al. fMRI data [8] than the approaches discussed in this paper. However, the range of possible further investigations in this direction is certainly far from exhausted, and, if better models are developed, the approach presented in this paper can be repeated to determine the new limiting factors in the data.

## References

[1] Martin A, Caramazza A (2003) Neuropsychological and neuroimaging perspectives on conceptual knowledge: An introduction. Cognitive Neuropsychology 20: 195–212. 20957570 10.1080/02643290342000050

[2] Tyler LK, Stamatakis EA, Dick E, Bright P, Fletcher P, et al (2003) Objects and their actions: Evidence for a neurally distributed semantic system. NeuroImage 18: 542–557. 12595206 10.1016/s1053-8119(02)00047-2

[3] Binder JR, Desai RH, Graves WW, Conant LL (2009) Where is the semantic system? A critical review and meta-analysis of 120 functional neuroimaging studies. Cerebral Cortex 19: 2767–2796. 19329570 10.1093/cercor/bhp055 PMC2774390

[4] Wang J, Conder JA, Blitzer DN, Shinkareva SV (2010) Neural representation of abstract and concrete concepts: A meta-analysis of neuroimaging studies. Human Brain Mapping 31: 1459–1468. 20108224 10.1002/hbm.20950 PMC6870700

[5] Binder JR, Desai RH (2011) The neurobiology of semantic memory. Trends in Cognitive Sciences 15: 527–536. 22001867 10.1016/j.tics.2011.10.001 PMC3350748

[6] Haxby JV, Gobbini MI, Furey ML, Ishai A, Schouten JL, et al (2001) Distributed and overlapping representations of faces and objects in ventral temporal cortex. Science 293: 2425–2430. 11577229 10.1126/science.1063736

[7] Cox DD, Savoy RL (2003) Functional magnetic resonance imaging (fMRI) “brain reading”: Detecting and classifying distributed patterns of fMRI activity in human visual cortex. NeuroImage 19: 261–270. 12814577 10.1016/s1053-8119(03)00049-1

[8] Mitchell TM, Shinkareva SV, Carlson A, Chang K-M, Malave VL, et al (2008) Predicting human brain activity associated with the meanings of nouns. Science 320: 1191–1195. 18511683 10.1126/science.1152876

[9] Lund K, Burgess C (1996) Producing high-dimensional semantic spaces from lexical co-occurrence. Behavior Research Methods, Instruments & Computers 28: 203–208.

[10] Patel M, Bullinaria JA, Levy JP (1997) Extracting semantic representations from large text corpora. In: Bullinaria JA, Glasspool DW, Houghton G, editors, Fourth Neural Computation and Psychology Workshop: Connectionist Representations. London: Springer. pp. 199–212.

[11] Landauer TK, Dumais ST (1997) A solution to Plato's problem: The Latent Semantic Analysis theory of acquisition, induction and representation of knowledge. Psychological Review 104: 211–240.

[12] Bullinaria JA, Levy JP (2007) Extracting semantic representations from word co-occurrence statistics: A computational study. Behavior Research Methods 39: 510–526. 17958162 10.3758/bf03193020

[13] Bullinaria JA, Levy JP (2012) Extracting semantic representations from word co-occurrence statistics: Stop-lists, Stemming and SVD. Behavior Research Methods 44: 890–907. 22258891 10.3758/s13428-011-0183-8

[14] Palatucci M, Hinton G, Pomerleau D, Mitchell TM (2009) Zero-shot learning with semantic output codes. Neural Information Processing Systems 22: 1410–1418.

[15] Devereux B, Kelly C, Korhonen A (2010) Using fMRI activation to conceptual stimuli to evaluate methods for extracting conceptual representations from corpora. In: Proceedings of the First Workshop on Computational Neurolinguistics. Stroudsburg, PA: ACL. pp. 70–78.

[16] Jelodar AB, Alizaseh M, Khadevi S (2010) WordNet based features for predicting brain activity associated with meanings of nouns. In: Proceedings of the First Workshop on Computational Neurolinguistics. Stroudsburg, PA: ACL. pp. 18–26.

[17] Pereira F, Botvinick M, Detre G (2010) Learning semantic features for fMRI data from definitional text. In: Proceedings of the First Workshop on Computational Neurolinguistics. Stroudsburg, PA: ACL. pp. 1–9.

[18] Levy JP, Bullinaria JA (2012) Using enriched semantic representations in predictions of human brain activity. In: Davelaar EJ, editor, Connectionist Models of Neurocognition and Emergent Behavior: From Theory to Applications. Singapore: World Scientific. pp. 292–308.

[19] Murphy B, Talukdar P, Mitchell T (2012) Selecting corpus-semantic models for neurolinguistic decoding. In: *SEM 2012: The First Joint Conference on Lexical and Computational Semantics. pp. 114–123.

[20] Pereira F, Botvinick M, Detre G (2013) Using Wikipedia to learn semantic feature representations of concrete concepts in neuroimaging experiments. Artificial Intelligence 194: 240–252. 23243317 10.1016/j.artint.2012.06.005 PMC3519435

[21] Bullinaria JA (2008) Semantic categorization using simple word co-occurrence statistics. In: Baroni M, Evert S, Lenci A, editors, Proceedings of the ESSLLI Workshop on Distributional Lexical Semantics. Hamburg, Germany: ESSLLI. pp. 1–8.

[22] Just MA, Cherkassky VL, Aryal S, Mitchell TM (2010) A neurosemantic theory of concrete noun representation based on the underlying brain codes. PLoS ONE 5(1): e8622. 20084104 10.1371/journal.pone.0008622 PMC2797630

[23] Zhao Y, Karypis G (2001) Criterion functions for document clustering: Experiments and analysis. Technical Report TR #01-040, Department of Computer Science, University of Minnesota Available: https://wwws.cs.umn.edu/tech_reports_upload/tr2001/01-040.pdf. Accessed 2013 Feb 22.

[24] Karypis G (2003) CLUTO: A Clustering Toolkit (Release 2.1.1). Technical Report: #02-017, Department of Computer Science, University of Minnesota Available from the CLUTO web-site: http://glaros.dtc.umn.edu/gkhome/views/cluto. Accessed 2013 Feb 22.

[25] Brants T, Franz A (2006) Web 1T 5-gram Version 1. Linguistic Data Consortium, Philadelphia Available: http://www.ldc.upenn.edu/Catalog/CatalogEntry.jsp?catalogId=LDC2006T13. Accessed 2013 February 22.

[26] Baroni M, Bernardini S, Ferraresi A, Zanchetta E (2009) The WaCky wide web: A collection of very large linguistically processed web-crawled corpora. Language Resources and Evaluation 43: 209–226 Corpus web-site: http://wacky.sslmit.unibo.it/doku.php. Accessed 2013 February 22.

[27] Manning CD, Schütze H (1999) Foundations of Statistical Natural Language Processing. Cambridge, MA: MIT Press.

[28] French RM, Labiouse C. (2002) Four problems with extracting human semantics from large text corpora. In: Proceedings of the Twenty-fourth Annual Conference of the Cognitive Science Society. Mahwah, NJ: Lawrence Erlbaum Associates. pp. 316–322.

[29] Erk K (2010). What Is Word Meaning, Really? (And How Can Distributional Models Help Us Describe It?). In: Proceedings of the 2010 Workshop on GEometrical Models of Natural Language Semantics. ACL. pp. 17–26.

[30] Caron J (2001) Experiments with LSA scoring: Optimal rank and basis. In: Berry MW, editor, Computational Information Retrieval. Philadelphia, PA: SIAM. pp. 157–169.

[31] Wang J, Baucom LB, Shinkareva SV (2012) (in press) Decoding abstract and concrete concept representations based on single-trial fMRI data. Human Brain Mapping 33 10.1002/hbm.21498 PMC6870364 23568269

[32] Raizada RDS, Connolly AC (2012) What makes different people's representations alike: Neural similarity space solves the problem of across-subject fMRI decoding. Journal of Cognitive Neuroscience 24: 868–877. 22220728 10.1162/jocn_a_00189

[33] Shinkareva SV, Malave VL, Mason RA, Mitchell TM, Just MA (2011) Commonality of neural representations of words and pictures. NeuroImage 54: 2418–2425. 20974270 10.1016/j.neuroimage.2010.10.042

[34] Raposo A, Moss HE, Stamatakis EA, Tyler LK (2009) Modulation of motor and premotor cortices by actions, action words and action sentences. Neuropsychologia 47: 388–396. 18930749 10.1016/j.neuropsychologia.2008.09.017

[35] Mahon BZ, Caramazza A (2010) Judging semantic similarity: An event-related fMRI study with auditory word stimuli. Neuroscience 169: 279–286. 20412836 10.1016/j.neuroscience.2010.04.029 PMC2908262

[36] Liu H, Palatucci M, Zhang J (2009) Blockwise coordinate descent procedures for the multi-task lasso, with applications to neural semantic basis discovery. In: Proceedings of the 26th Annual International Conference on Machine Learning. New York, NY: ACM Press. pp. 649–656.

[37] Chen X, He J, Lawrence R, Carbonell JG (2012) Adaptive multi-task sparse learning with an application to fMRI study. In: Proceedings of the Twelfth SIAM International Conference on Data Mining. Philadelphia, PA: SIAM. pp. 212–223.

